# Therapy with lopinavir/ritonavir and hydroxychloroquine is associated with acute kidney injury in COVID-19 patients

**DOI:** 10.1371/journal.pone.0249760

**Published:** 2021-05-11

**Authors:** Johanna Schneider, Bernd Jaenigen, Dirk Wagner, Siegbert Rieg, Daniel Hornuss, Paul M. Biever, Winfried V. Kern, Gerd Walz

**Affiliations:** 1 Department of Medicine IV, Faculty of Medicine, Medical Center, University of Freiburg, Freiburg, Germany; 2 Department of General and Digestive Surgery, Faculty of Medicine, Medical Center, University of Freiburg, Freiburg, Germany; 3 Department of Medicine II, Division of Infectious Diseases, Faculty of Medicine, Medical Center, University of Freiburg, Freiburg, Germany; 4 Department of Medicine III (Interdisciplinary Medical Intensive Care), Medical Center–University of Freiburg, Faculty of Medicine, University of Freiburg, Freiburg, Germany; 5 Department of Cardiology and Angiology I, Heart Center Freiburg University, Medical Center–University of Freiburg, Faculty of Medicine, University of Freiburg, Freiburg, Germany; National Yang-Ming University, TAIWAN

## Abstract

**Background:**

Acute kidney injury (AKI) is an independent risk factor for mortality, which affects about 5% of hospitalized coronavirus disease-2019 (COVID-19) patients and up to 25–29% of severely ill COVID-19 patients. Lopinavir/ritonavir and hydroxychloroquine show *in vitro* activity against severe acute respiratory syndrome coronavirus 2 (SARS-CoV-2) and have been used for the treatment of COVID-19. Both, lopinavir and hydroxychloroquine are metabolized by cytochrome P450 (CYP) 3A4. The impact of a triple therapy with lopinavir/ritonavir and hydroxychloroquine (triple therapy) on kidney function in COVID-19 is currently not known.

**Methods:**

We retrospectively analyzed both non-ICU and ICU patients with COVID-19 receiving triple therapy for the incidence of AKI. Patients receiving standard therapy served as a control group. All patients were hospitalized at the University Hospital of Freiburg, Germany, between March and April 2020. A matched-pair analysis for the National Early Warning Score (NEWS) 2 was performed to control for the severity of illness among non-intensive care unit (ICU) patients.

**Results:**

In non-ICU patients, the incidence of AKI was markedly increased following triple therapy (78.6% vs. 21.4% in controls, p = 0.002), while a high incidence of AKI was observed in both groups of ICU patients (triple therapy: 80.0%, control group: 90.5%). ICU patients treated with triple therapy showed a trend towards more oliguric or anuric kidney injury. We also observed a linear correlation between the duration of the triple therapy and the maximum serum creatinine level (p = 0.004, R^2^ = 0.276, R = 0.597).

**Conclusion:**

Triple therapy is associated with an increase in the incidence of AKI in non-ICU COVID-19 patients. The underlying mechanisms may comprise a CYP3A4 enzyme interaction, and may be relevant for any future therapy combining hydroxychloroquine with antiviral agents.

## Introduction

Acute kidney injury (AKI) is a frequent complication in about 5% of hospitalized patients with coronavirus disease-2019 (COVID-19) and an independent risk factor for in-hospital death [1]. 43.9% of COVID-19 patients exhibit proteinuria and 26.7% hematuria, which are both independent risk factors for mortality [1]. The incidence of AKI is higher in severely ill patients with observational studies from Wuhan, China, reporting AKI in 25–29% critically ill patients [2, 3]. A similar incidence was reported from Washington State, USA, with 19.1% of critically ill patients [4]. Renal replacement therapy (RRT) was required by 5–19% [2, 5]. Potential mechanisms causing AKI in COVID-19 comprise cytokine release, hemodynamic changes, and direct viral cytotoxicity [6]. Autopsy studies mainly report acute tubular injury [7, 8]. In a previous study reporting the results of 26 autopsies, 9 patients showed diffuse proximal tubule injury with clusters of coronavirus particles in the tubular epithelium and podocytes as observed in electron micrographs [8].

No specific therapy for COVID-19 was available when treatment of COVID-19 patients was started at the University Hospital of Freiburg, Germany, in March 2020. The combined protease inhibitor lopinavir/ritonavir is used for the treatment of HIV infection and has both *in vitro* activity against SARS-CoV-2 [9] and *in vivo* activity against Middle East respiratory syndrome coronavirus (MERS-CoV) as shown in animal studies [10]. The autophagy inhibitor hydroxychloroquine is used for the treatment of malaria and several autoimmune disorders and exhibits *in vitro* activity against SARS-CoV-2 [11]. The evidence for the efficacy of both drugs given alone has been very limited, based on studies published until July 2020 [12]. Based on early reports the first patients with severe COVID-19 at the University Hospital of Freiburg were treated with a combination of lopinavir/ritonavir and hydroxychloroquine (triple therapy).

Known adverse effects for lopinavir are mostly gastrointestinal, including nausea, vomiting, and diarrhea. Common side effects of hydroxychloroquine include abnormal heart rhythms, such as QT interval prolongation and ventricular tachycardia [13]. Both drugs are metabolized by cytochrome P450 (CYP) 3A4 in the liver, thus a drug interaction is plausible [14–16]. Hydroxychloroquine is N-dealkylated by CYP3A4 to the biologically active metabolite desethylhydroxychloroquine and the inactive metabolites desethylchloroquine and bidesethylchloroquine [14, 15]. The oral bioavailability of lopinavir is low due to its rapid metabolism mainly by CYP3A4 and it is therefore co-administered with the CYP3A4 inhibitor ritonavir [16–20].

After treating the first COVID-19 patients at our hospital with triple therapy, the impression emerged that the incidence of acute kidney injury was increased. Thus, data available from these patients were analyzed and are presented in this report. The goal of the present study was to determine the impact of the triple therapy on the development of AKI in COVID-19 patients and to guide treatment decisions for future patients. Preliminary results showed an increased incidence of acute kidney injury in the triple therapy group compared to a control group, which was possibly caused by a CYP3A4 drug interaction. Therefore, the triple therapy regime was stopped immediately.

## Materials and methods

### Study design

A retrospective analysis of hospitalized patients with COVID-19 at the University Hospital of Freiburg, Germany, in March and April 2020 was performed. Inclusion criteria were hospitalization, a positive test for SARS-CoV2 and being symptomatic for COVID-19. Asymptomatic patients were excluded. The diagnosis of SARS-CoV-2 infection was established via RT-PCR of a nasopharyngeal or oropharyngeal swab specimen. The primary objective was to determine the incidence of acute kidney injury. The following secondary outcomes were evaluated: need of renal replacement therapy, admission to the ICU, invasive ventilation, extracorporal membrane oxygenation and mortality.

**Fig 1:**
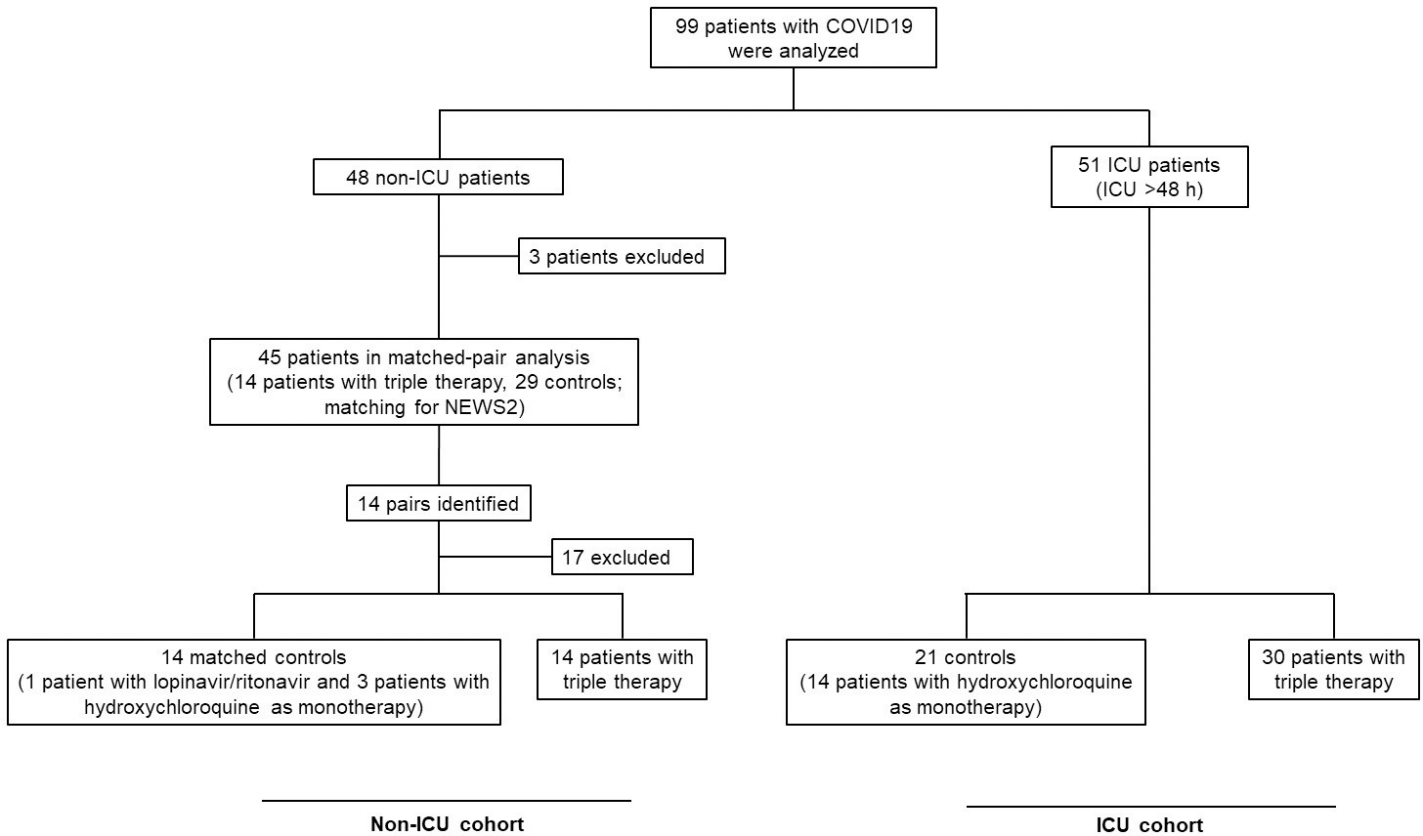
Study design. COVID-19, coronavirus disease-2019; ICU, intensive care unit; NEWS2, National Early Warning Score 2; triple therapy, therapy with lopinavir/ritonavir and hydroxychloroquine.

99 patients who were treated for COVID-19 were identified by screening patient records of ICU and non-ICU patients (Fig 1). Patients who were at the ICU for less than 48h were defined as non-ICU patients. This definition excludes patients that were admitted to the ICU for interventional procedures, such as pleural puncture or insertion of a central venous line. 48 patients were defined as non-ICU patients. Two patients were asymptomatic. These patients were therefore excluded from the analysis. One patient was at our hospital for a short period of time with a duration of stay <48h at the ICU and a similar duration of stay at the ICU and the normal ward. This patient was also excluded from the analysis. Patients in the ICU cohort were typically admitted to the intensive care unit via the emergency room or via the normal ward; a subgroup of patients was directly transferred to the ICU from other hospitals.

14 patients of the remaining 45 patients received a triple therapy with lopinavir/ritonavir (Kaletra^®^, AbbVie, North Chicago, IL, USA) and hydroxychloroquine (Quensyl^®^, Sanofi-Aventis, Frankfurt, Germany). Lopinavir/ritonavir was administered at a dose of 400/100 mg twice daily for 5 days and hydroxychloroquine at a dose of 400 mg twice daily during the initial 24 h followed by 200 mg twice daily for another six days, based on the study by Yao et al. [11]. Since patients of the non-ICU cohort with triple therapy were more severely affected by SARS-CoV-2 than the control group, a matched-pair analysis was performed. Matched pairs were identified by a 1:1 matching with SPSS Statistics 25^®^ software (IBM Corp., Armonk, NY) for the National Early Warning Score (NEWS) 2 (match tolerance of ≤1). The NEWS2 comprises the following criteria: respiratory rate, oxygen saturation, oxygen use, systolic blood pressure, heart rate, consciousness and temperature. We decided to match for NEWS2, since this score is a predictive marker for the outcome and the severity of illness in COVID-19 patients [21]. 14 pairs were identified and analyzed. One patient of the control group received lopinavir/ritonavir and three received hydroxychloroquine as a monotherapy (Fig 1).

The second patient cohort consists exclusively of COVID-19 patients treated in the ICU for more than 48h. 51 patients were identified. Triple therapy was administered to 30 patients, 21 patients served as a control group. 14 patients of the control group were treated with hydroxychloroquine, but not lopinavir/ritonavir, 7 patients received neither lopinavir/ritonavir nor hydroxychloroquine (Fig 1). Since the severity of illness indicated by the Simplified Acute Physiology Score (SAPS) 2 was similar between groups (Table 4), no matching was performed. The percentage of missing data is indicated in the Tables 1–5.

**Table 1:** Characteristics of non-ICU patients treated with a triple therapy (lopinavir/ritonavir and hydroxychloroquine) compared to a control group.

Parameter		Control group	Triple therapy (lopinavir/ritonavir and hydroxychloroquine)	p-value
		n = 14	n = 14	
Hydroxychloroquine monotherapy, n (%)		3 (21.4)		
Lopinavir/ritonavir monotherapy, n (%)		1 (7.1)		
NEWS2, mean ± SD		6.7 ± 2.2	6.5 ± 2.2	0.797
Sex (male), n (%)		7 (50.0)	9 (64.3)	0.704
Age (years), median (IQR)		70.5 (21.0)	67.0 (26.5)	0.940
Median length of hospital stay (days), median (IQR) (3.6% data missing)		13.0 (13.3)	18.0 (16.8)	0.080
Discharge from hospital, n (%)		12 (85.7)	13 (92.9)	1.000
Body mass index (kg/m^2^), median (IQR) (32.1% data missing)		23.4 (7.7)	26.7 (8.1)	0.864
Smoking history, n (%)		1 (7.1)	4 (28.6)	0.326
Number of coexisting disorders, mean ± SD		2.1 ± 1.6	2.9 ± 1.2	0.148
	Cardiac, n (%)	6 (42.9)	10 (71.4)	0.252
	Pulmonary, n (%)	1 (7.1)	8 (57.1)	0.013*
	Hepatic, n (%)	2 (14.3)	1 (7.1)	1.000
	Cancer, n (%)	2 (14.3)	2 (14.3)	1.000
	Hemic disease, n (%)	2 (14.3)	3 (21.4)	1.000
	Diabetes, n (%)	3 (21.4)	2 (14.3)	1.000
	Chronic kidney disease, n (%)	3 (21.4)	5 (35.7)	0.678
	Hypertension, n (%)	7 (50)	6 (42.9)	1.000
	Dementia, n (%)	1 (7.1)	1 (7.1)	1.000
	Cerebrovascular, n (%)	3 (21.4)	3 (21.4)	1.000
Antibiotics, n (%)		7 (50.0)	4 (28.6)	0.440
Immunosuppressive therapy, n (%)		1 (7.1)	2 (14.3)	1.000
Fever (>38°C), n (%)		13 (92.9)	14 (100.0)	1.000
Hypotension (systolic blood pressure < 100 mmHg), n (%)		6 (42.9)	8 (57.1)	0.706
Maximum oxygen supply for at least 12 h (L/min), median (IQR)		0 (3.0)	2.0 (5.3)	0.177
C-reactive protein (mg/L), median (IQR)		52.8 (102.6)	115.5 (249.5)	0.284
Interleukin-6 (pg/mL), median (IQR)		59.9 (90.1)	184.5 (249.5)	0.032*
Lactate dehydrogenase (U/L), mean ± SD (3.6% data missing)		416.1 ± 154.1	374.3 ± 110.4	0.428

NEWS2, National Early Warning Score; IQR, interquartile range; SD, standard deviation. Note that data, which are normally distributed (Shapiro-Wilk test) are presented as mean ± standard deviation and data not normally distributed are presented as median (interquartile range); * p<0.05.

**Table 2:** Acute kidney injury and outcome in non-ICU patients.

Parameter		Control group	Triple therapy (lopinavir/ritonavir and hydroxychloroquine)	p-value
		n = 14	n = 14	
Baseline serum creatinine (mg/dL), mean ± SD		0.9 ± 0.4	1.0 ± 0.3	0.629
Maximum serum creatinine (mg/dL), median (IQR)		0.9 (0.6)	1.4 (0.9)	0.015*
Delta serum creatinine (mg/dL), median (IQR)		0.1 (0.3)	0.5 (0.6)	0.003*
AKI, n (%)		2 (14.3)	11 (78.6)	0.002*
	AKI I, n (%)	2 (14.3)	8 (57.1)	0.002*
	AKI II, n (%)	0 (0)	2 (14.3)	0.003*
	AKI III, n (%)	0 (0)	1 (7.1)	0.002*
Urine analysis				
	Hematuria, median (IQR), (23.1% data missing)	2.0	1.0 (2.0)	0.386
	Proteinuria, median (IQR), (23.1% data missing)	1.0	1.0 (1.5)	0.772
	Leucocyturia, median (IQR), (23.1% data missing)	3.0	0 (0)	0.035*
Duration between first day of symptoms and AKI (days), mean ± SD, (7.1% data missing)		5.0	6.1 ± 5.6	0.857
Duration between first positive test and AKI (days), mean ± SD		2.5 ± 2.1	3.1 ± 4.2	0.852
Duration of triple therapy (days), mean ± SD			4.6 ± 0.9	
Duration between start of triple therapy and AKI (days), mean ± SD			1.7 ± 3.1	
Clinical characteristics at day before AKI				
	Systolic blood pressure (mmHg), mean ± SD	105 ± 7.1	121.7 ± 21	0.302
	Diastolic blood pressure (mmHg), mean ± SD	57.5 ± 3.5	60.7 ± 15.4	0.781
	Diarrhea, n (%)	0 (0)	1 (9,1)	1.000
	Fever, n (%)	1 (50)	8 (72,7)	1.000
Disease-related AKI		2 (100.0)	4 (36.4)	0.192
RRT, n (%)		0 (0.0)	0 (0.0)	1.000
Admission to ICU (< 48 h), n (%)		0 (0)	2 (14.3)	0.481
Invasive ventilation, n (%)		0 (0)	0 (0)	1.000
Mortality, n (%)		2 (14.3)	3 (21.4)	1.000

Hematuria, leucocyturia and proteinuria were measured semi-quantitatively by standard urine dipstick analysis. The values refer to a grading from negative to 3+ in case of proteinuria and leucocyturia and from negative to 4+ in hematuria. Urine analysis was performed for patients with acute kidney injury, therefore data missing in urine analysis refer to the number of patients with acute kidney injury. For the control group only one urine analysis was available. Disease-related AKI was defined as a simultaneous increase of creatinine and procalcitonin.

AKI, acute kidney injury; ICU, intensive care unit; IQR, interquartile range; RRT, renal replacement therapy; SD, standard deviation; triple therapy, therapy with lopinavir/ritonavir and hydroxychloroquine. Note that data, which are normally distributed (Shapiro-Wilk test) are presented as mean ± standard deviation and data not normally distributed are presented as median (interquartile range);

* p<0.05.

**Table 3:** Multivariable analysis for acute kidney injury adjusted for NEWS2.

Variable	Odds ratio Variable	95% CI	p-value	Odds ratio Triple therapy	95% CI	p-value
Age	1.1	1.0–1.1	0.167	54.6	3.3–911.2	0.005*
Sex (male)	2.0	0.2–18.1	0.550	38.4	3.4–439.8	0.003*
Body mass index	0.9	0.7–1.2	0.605	14.1	1.4–140.5	0.024*
Number of coexisting disorders	3.1	1.1–8.8	0.035*	47.9	2.3–993.0	0.012*
Pulmonary disease	6.3	0.5–88.6	0.170	16.1	1.4–182.6	0.025*
Antibiotics	1.0	0.1–8.7	0.974	33.6	3.0–371.4	0.004*
Immunosuppressive therapy	1.0	0.1–21.5	0.987	33.4	3.2–347.9	0.003*
Hypotension	1.4	0.2–11.5	0.778	35.1	3.3–374.5	0.003*
Maximum oxygen supply for at least 12 h	1.1	0.8–1.4	0.654	34.1	3.2–367.1	0.004*
Interleukin 6	1.0	1.00–1.03	0.084	25.1	1.7–371.1	0.019*
C-reactive protein	1.0	1.00–1.02	0.579	31.4	2.9–339.1	0.005*
Lactate dehydrogenase	1.0	1.00–1.01	0.668	35.8	2.8–459.2	0.006*

NEWS 2, National Early Warning Score; triple therapy, therapy with lopinavir/ritonavir and hydroxychloroquine

**Table 4:** Characteristics of ICU patients treated with a triple therapy (lopinavir/ritonavir and hydroxychloroquine) compared to a control group.

Parameter		Control group	Triple therapy (lopinavir/ritonavir and hydroxychloroquine)	p-value
		n = 21	n = 30	
Hydroxychloroquine monotherapy		14 (66.7)		
Sex (male), n (%)		17 (81.0)	21 (70.0)	0.518
Age (years), mean ± SD		64.2 ± 14.1	62.1 ± 9.4	0.525
Median length of ICU stay (days), mean ± SD		14.4 ± 6.6	19.3 ± 10.1	0.056
Discharge from hospital, n (%)		8 (38.1)	22 (73.3)	0.020*
Body mass index (kg/m^2^), median (IQR) (45.1% data missing)		27.8 (7.9)	29.4 (5.9)	0.564
Number of coexisting disorders, median (IQR)		2.0 (2.0)	1.0 (2.0)	0.171
	Cardiac, n (%)	6 (28.6)	10 (33.3)	0.768
	Pulmonary, n (%)	4 (19.1)	6 (20.0)	1.000
	Hepatic, n (%)	0 (0)	1 (3.3)	1.000
	Cancer, n (%)	1 (4.8)	4 (13.3)	0.391
	Hemic, n (%)	6 (28.6)	2 (6.7)	0.052
	Diabetes, n (%)	4 (19.1)	5 (16.7)	1.000
	Chronic kidney disease, n (%)	7 (33.3)	3 (10.0)	0.070
	Hypertension, n (%)	9 (42.9)	14 (46.7)	1.000
	Dementia, n (%)	2 (9.5)	0 (0.0)	0.165
	Cerebrovascular, n (%)	4 (19.0)	0 (0.0)	0.024*
SAPS 2, median (IQR)		46.0 (13.0)	48.0 (8.5)	0.843
Invasive ventilation, n (%)		17 (81.0)	28 (93.3)	0.214
	PaO_2_ (mmHg), median (IQR)	72.0 (11.5)	68.5 (12.5)	0.270
	FiO_2_ (%), median (IQR)	40.0 (10.0)	40.0 (8.8)	0.601
	PaO_2_/FiO_2_, median (IQR)	180.0 (51.5)	161.5 (45.3)	0.350
Extracorporeal membrane oxygenation, n (%)		7 (33.3)	10 (33.3)	1.000
Vasopressor use, n (%)		14 (66.7)	27 (90.0)	0.070
C-reactive protein (mg/L), mean ± SD		271.0 ± 107.5	298.4 ± 105.2	0.368
Interleukin-6 (pg/mL), median (IQR) (2.0% data missing)		339 (4198)	466.5 (1650.7)	0.770
Procalcitonin (ng/mL), median (IQR)		3.9 (19.3)	5.1 (12.8)	0.478
D-dimer (mg/L), median (IQR) (13.7% data missing)		7.6 (32.9)	21.4 31.6)	0.698
Lactate dehydrogenase (U/L), median (IQR		496.0 (367.0)	686.0 (463.0)	0.041*
Creatine kinase (U/L), median (IQR) (3.9% data missing)		239.0 (1380.0)	651.5 (1075)	0.402
Aspartate aminotransferase, (U/L), median (IQR)		112.0 (204.0)	111.5 (82.0)	0.236
Alanine aminotransferase (U/L), median (IQR)		58.0 (51.0)	61.0 (34.0)	0.170

FiO_2_, Fraction of inspired oxygen; ICU, intensive care unit; PaO_2_, Arterial partial pressure of oxygen; SAPS 2, Simplified Acute Physiology Score SAPS 2; SD, standard deviation. Note that data, which are normally distributed (Shapiro-Wilk test) are presented as mean ± standard deviation and data not normally distributed are presented as median (interquartile range);

* p<0.05.

**Table 5:** Acute kidney injury in ICU patients.

Parameter		Control group	Triple therapy (lopinavir/ritonavir and hydroxychloroquine)	p-value
		n = 21	n = 30	
Baseline serum creatinine (mg/dL), median (IQR) (9.8% data missing)		1.0 (0.4)	0.8 (0.3)	0.059
Maximum serum creatinine (mg/dL), median (IQR)		3.3 (3.3)	3.1 (5.5)	0.776
Delta serum creatinine (mg/dL), median (IQR), (9.8% data missing)		2.0 (2.7)	2.4 (4.6)	0.714
AKI, n (%)		19 (90.5)	24 (80.0)	0.445
	AKI I, n (%)	7 (33.3)	6 (20.0)	0.338
	AKI II, n (%)	3 (14.3)	2 (6.7)	0.637
	AKI III, n (%)	9 (42.9)	16 (53.3)	0.572
Urine analysis				
	Hematuria, median (IQR), (20.9% data missing)	2.5 (3.0)	2.5 (1.0)	0.704
	Proteinuria, median (IQR), (20.9% data missing)	1.5 (1.0)	1.5 (1.0)	1.000
	Leucocyturia, median (IQR), (20.9% data missing)	0.5 (2.0)	0.0 (1.0)	0.014*
	Muddy brown casts, n (%) (34.9% data missing)	5 (55.6)	11 (57.9)	1.000
Duration between first day of symptoms and AKI (days), mean ± SD (34.9% data missing)		11.9 ± 8.8	10.0 ± 3.9	0.433
Duration between admission to ICU and AKI (days), mean ± SD		3.1 ± 5.5	2.8 ± 4.3	0.862
Duration of triple therapy (days), mean ± SD			3.0 ± 2.9	
Duration between start of triple therapy and AKI (days), mean ± SD			2.4 ± 4.0	
Diuresis: an-/oliguric, n (%)		5 (23.8)	12 (40.0)	0.366
Renal replacement therapy (RRT), n (%)		6 (28.6)	12 (40.0)	0.553
Duration between first day of symptoms and start of RRT (days), mean ± SD (38.9% data missing)		11.0 ± 7.1	16.4 ± 5.2	0.232
Duration between admission to ICU and start of RRT (days), mean ± SD		9.3 ± 7.3	6.8 ± 4.2	0.353
Mortality, n (%)		3 (14.3)	10 (34.5)	0.193

Hematuria, leucocyturia and proteinuria were measured semi-quantitatively by standard urine dipstick analysis. The values refer to a grading from negative to 3+ in case of proteinuria and leucocyturia and from negative to 4+ in hematuria. Urine analysis was performed for patients with acute kidney injury, therefore data missing in urine analysis refer to the number of patients with acute kidney injury.

AKI, acute kidney injury; ICU, intensive care unit; IQR, interquartile range; RRT, renal replacement therapy; triple therapy, combined therapy with lopinavir/ritonavir and hydroxychloroquine. Note that data, which are normally distributed (Shapiro-Wilk test) are presented as mean ± standard deviation and data not normally distributed are presented as median (interquartile range);

* p<0.05.

### Definitions

Acute kidney injury (AKI) was defined according to the KDIGO 2012 criteria [22]. AKI stage I was defined as an increase in serum creatinine 1.5–1.9 times baseline, AKI stage II was defined as an increase 2.0–2.9 times baseline and AKI stage III was defined as an increase 3 times baseline or ≥4.0 mg/dL increase or the initiation RRT. Urine output, which is one of the KDIGO AKI criteria, was not taken into account as it was not measured on a regular basis in non-ICU patients. The baseline serum creatinine was defined as the nadir creatinine at the time of admission to hospital or, if available, previously measured serum creatinine values.

### Statistical analysis

Clinical data were collected from historical records. SPSS Statistics 25^®^ software was used for statistical analysis. Continuous variables were expressed as mean ± standard deviation. Fisher’s exact tests were performed on categorical variables. Shapiro-Wilk test was performed to test whether continuous variables were normally distributed. In case of normal distribution, student’s t-tests were performed and data are presented as mean ± standard deviation (SD). If continuous variables were not normally distributed data are presented as median and interquartile range (IQR) and an independent t-test was performed after log transformation. Multivariable logistic regression analysis was used to identify variables associated with the occurrence of acute kidney injury. Odds ratios (OR) and 95% confidence intervals (CI) were calculated by exponentiation of logistic regression coefficients. When calculating the logistic regression, triple therapy, NEWS2 and an additional variable to be examined were specified as independent variables and the odds ratio calculated for each variable. This approach was chosen to take into account that the cohort was not selected at random but by a matched-pair analysis. As matching was performed for the NEWS2, this score was included as an independent variable. A linear regression analysis was performed to test the impact of the duration of triple therapy on the maximum serum creatinine level using GraphPad Prism 6^®^ (GraphPad Software, San Diego, CA, USA), followed by a Spearman´s rank correlation. All tests were 2-tailed; a p-value <0.05 was considered statistically significant.

The study was approved by the ethics committee of the University of Freiburg Medical Center, Germany (protocol number 276/20) and is registered at the DRKS (Deutsches Register klinischer Studien, DRKS00021658). The ethics committee waived the requirement for informed consent.

## Results

### Non-ICU patients

The triple therapy group and the control group of the non-ICU cohort consisted of 14 patients each. Groups did not differ in terms of age, sex, median length of hospital stay or body mass index (Table 1). The number of coexisting disorders was similar with 2.9 ± 1.2 in the triple therapy treated group and 2.1 ± 1.6 in the control group (p = 0.148, Table 1). More patients in the triple therapy treated group had preexisting pulmonary disease (57.1% vs. 7.1% in the control group, p = 0.013, Table 1); all other preexisting diseases were evenly distributed. The maximum oxygen supply needed for at least 12 h was similar (p = 0.177, Table 1). A similar number of patients in both group showed hypotension and fever. The maximum interleukin-6 level was higher in the triple therapy group (184.5 (249.5) pg/ml vs. 59.5 (90.1) pg/mL in the control group, p = 0.032, Table 1).

**Fig 2:**
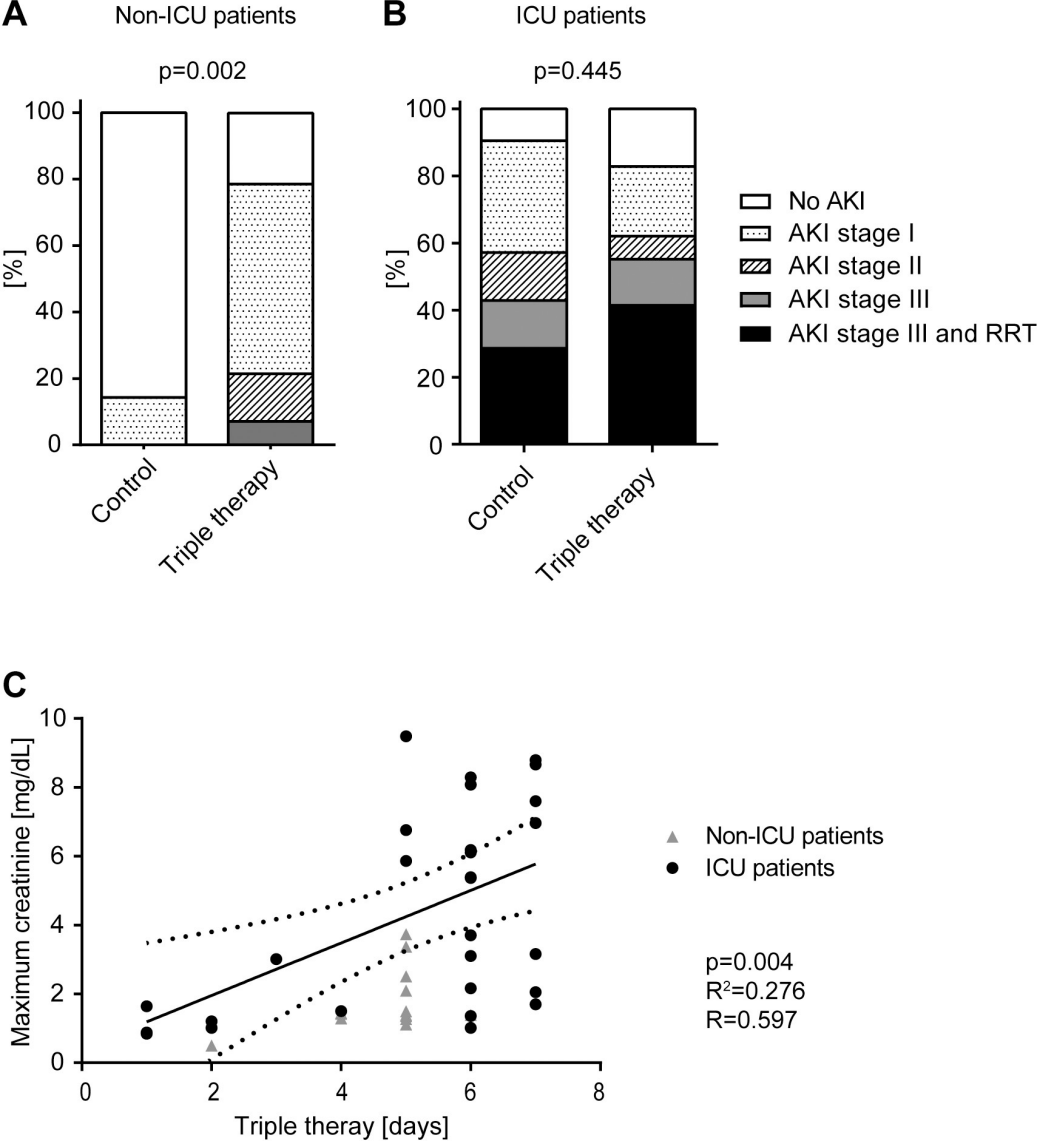
Lopinavir/ritonavir and hydroxychloroquine (triple therapy) are associated with an increase in the incidence of Acute Kidney Injury (AKI). Association between triple therapy and AKI (A) in non-intensive care unit (ICU) patients and (B) ICU patients. P-values refer to the total number of AKI; RRT, renal replacement therapy. (C) Association between triple therapy and the maximum serum creatinine value.

The baseline serum creatinine level did not differ between groups. Importantly, the incidence of acute kidney injury was significantly increased in the triple therapy treated group (78.6% vs. 14.3%, p = 0.002, Table 2 and Fig 2A). AKI occurred 6.1 days after the first symptoms in the triple therapy group and after 5.0 days in the control group (p = 0.857, Table 2), and 2.5 days after the first positive test for SARS-CoV-2 in the control group vs. 3.1 days in the triple therapy group (p = 0.852, Table 2). Dipstick urine analysis showed slight hematuria and proteinuria in both groups (Table 2). Clinical characteristics prior to the onset of acute kidney injury showed no difference in terms of blood pressure, diarrhea and fever. 36.4% of patients with AKI in the triple therapy group and all patients with AKI in the control group showed a parallel increase in serum creatinine and procalcitonin (p = 0.192; Table 2), which was classified as “disease-related AKI”. None of the patients received nephrotoxic medication. None of the patients needed renal replacement therapy or invasive ventilation and the mortality rate did not differ between groups (Table 2).

We evaluated the influence of triple therapy and other factors like age, NEWS2, sex, body mass index, the number of coexisting disorders, pulmonary disease, antibiotics, immunosuppressive therapy, hypotension, the maximum oxygen supply, interleukin 6, C-reactive protein, and lactate dehydrogenase by a multivariable analysis. The analysis showed that triple therapy in general has a strong influence and only the number of coexisting disorders had an additional significant influence on the development of acute kidney injury (number of coexisting disorders: odds ratio 3.09, p = 0.035, Table 3).

### ICU patients

Among the 51 patients in the ICU cohort, 30 received triple therapy, 14 control patients received hydroxychloroquine monotherapy, and 7 received no antiviral therapy (Table 4). Groups did not differ in terms of sex, age, median length of ICU stay, number of coexisting disorders or inflammatory parameters, i.e. C-reactive protein, interleukin-6 and procalcitonin. The SAPS 2 was similar between groups (triple therapy group: 46.0 (13.0), control group: 48.0 (8.5), p = 0.843, Table 4). A similar number of patients needed invasive ventilation (control group: 81.0%, triple therapy group: 93.3%, p = 0.214, Table 4) or extracorporal membrane oxygenation (control group: 33.3%, triple therapy group: 33.3%, p = 1.000, Table 4). There was no difference in the fraction of inspired oxygen (FiO_2_), the arterial partial pressure of oxygen (PaO_2_) and the PaO_2_/FiO_2_ ratio between groups. We observed a trend towards a higher incidence of preexisting chronic kidney disease in the control group (control group: 33.3%, triple therapy group: 10.0%, p = 0.070, Table 4) and patients in the control group showed a trend towards a higher baseline serum creatinine (control group: 1.0 (0.4) mg/dL, triple therapy group: 0.8 (0.3) mg/dL, p = 0.059).

Almost all patients of the ICU cohort developed in-hospital AKI with 80% of patients with triple therapy and 90.5% of patients in the control group (p = 0.445, Table 5). 40% of patients with triple therapy and 23.8% of the control group developed oliguria or anuria (p = 0.366, Table 5) and 40% of patients with triple therapy and 28.6% of the control group needed RRT (p = 0.553, Table 5 and Fig 2B). Urine dipstick analysis indicated hematuria and proteinuria in both groups. Urine sediment analysis showed muddy brown casts and indicated acute tubular necrosis in more than 50% of both groups (p = 1.000, Table 5). AKI occurred after a median of 2.8 ± 4.3 days following admission to the ICU in the triple therapy group and after 3.1 ± 5.5 days in the control group (p = 0.862, Table 5).

A linear correlation between the duration of lopinavir/ritonavir and hydroxychloroquine therapy and the maximum serum creatinine value was observed in ICU and non-ICU patients (Fig 2C, R^2^ = 0.276, R = 0.597, p = 0.004), indicating a higher maximum serum creatinine value in patients with a longer duration of therapy.

## Discussion

Acute kidney injury in COVID-19 affects about 5% of hospitalized patients and about 25–29% of critically ill patients [1–3] with a high variety depending on the severity of illness. AKI was observed in about 50% of non-ICU patients in our cohort (Table 2), indicating that the analyzed non-ICU cohort was severely ill. Importantly, while AKI occurred in 14.3% of the untreated patients, the incidence increased to 78.6% in patients treated with lopinavir/ritonavir and hydroxychloroquine (p = 0.002, Table 2). Since the baseline characteristics in the non-ICU cohort were similar except for preexisting pulmonary diseases, we suspect that the higher incidence of AKI is most likely caused by the triple therapy. This is supported by the linear correlation observed between the duration of the triple therapy and the maximum serum creatinine value (R^2^ = 0.276, R = 0.597, p = 0.004, Fig 2C) and by a multivariable analysis showing a significant influence of triple therapy on the development of AKI (Table 3). Based on this analysis, lopinavir/ritonavir treatment of COVID-19 patients was immediately stopped at our institution.

Both, lopinavir and hydroxychloroquine are metabolized by the CYP3A4, which bears the risk of a drug interaction. Adverse events of lopinavir are mostly gastrointestinal [13]; however, the incidence of diarrhea before AKI was low in non-ICU patients with triple therapy. Thus, prerenal kidney injury due to diarrhea was an unlikely cause for the observed differences. Known renal side effects of lopinavir/ritonavir include a reduction of the glomerular filtration rate [23, 24] and proteinuria and glycosuria in human immunodeficiency virus (HIV)-positive patients [6]. A previous study reports a trend towards a higher incidence of biopsy-proven acute tubular injury [25]. Hydroxychloroquine rarely causes renal side effects, with cases reporting phospholipidosis, a histomorphologic change similar to Fabry nephropathy [26, 27]. In our patient cohort, urine analysis revealed hematuria and proteinuria (Tables 2 and 5) as previously described for COVID-19 patients [1]. Muddy brown casts were observed in ICU patients (Table 5) with no difference between the triple therapy treated group and the control group. In a retrospective analysis, 37.8% of COVID-19 patients showed adverse drug events, and 63.8% of these events were explained by the use of lopinavir/ritonavir [28]. The risk was increased with the number of co-administered drugs, but did not include the co-administration of hydroxychloroquine. Although the incidence of AKI was not increased following lopinavir/ritonavir monotherapy in COVID-19 patients in a randomized-controlled study [29], the results of the present study suggest that combining hydroxychloroquine with antiviral agents that are also metabolized by CYP3A4 is associated with an increased incidence of AKI. Only one case with AKI caused by a drug interaction with lopinavir/ritonavir has been reported [30]. In this case, AKI was most likely caused by hypotension, but not direct nephrotoxicity. In the present study, blood pressure before AKI was not different between groups (Table 1). No patient of this cohort received a known nephrotoxic medication, which could explain the difference in the incidence of AKI. Thus, AKI based on a drug interaction other than lopinavir/ritonavir/ hydroxychloroquine is unlikely.

Almost all ICU patients developed AKI with a non-significant trend towards a higher degree of AKI severity in triple therapy treated patients (AKI stage III: 53.3% vs. 42.9%, p = 0.572, Table 5). Of note, the control group showed a trend towards more patients with chronic kidney disease and a higher baseline serum creatinine, which is a risk factor for acute kidney injury [31, 32]. This provides a potential explanation for the similar incidence of AKI in the ICU cohort despite a potential harmful effect of the triple therapy.

Limitations of our study are related to its retrospective observational design, limited time frame, the difference in the C-reactive protein and interleukin-6 level between the non-ICU groups, multiple testing, and restriction to a single center. Based on the small study population, the clinical significance of this analysis should be interpreted with caution. Regarding COVID-19, the RECOVERY trial that tested high dose hydroxychloroquine stopped enrolling patients after an interim analysis in June showed no beneficial effects of this treatment in COVID-19 patients (RECOVERY Collaborative Group 2020). A preliminary analysis of these data indicated no increase in renal toxicity [33]. Both, hydroxychloroquine and lopinavir should no longer be prescribed for treatment of SARS-CoV-2 infection due to lack of convincing efficacy.

In summary, our study indicates that a triple therapy with lopinavir/ritonavir and hydroxychloroquine promotes AKI in COVID-19 patients, which might be relevant for any treatment strategies combining hydroxychloroquine with antiviral agents that utilize CYCP3A4 metabolism.
